# Exploring the Influential Factors Impacting the Provision of Family-Centered Care for Children with Cerebral Palsy in Saudi Arabia

**DOI:** 10.3390/children10121868

**Published:** 2023-11-29

**Authors:** Ahmad Abdullah Alharbi, Abdulaziz Aoudh Albalwi

**Affiliations:** Department of Physical Therapy, Faculty of Applied Medical Sciences, University of Tabuk, Tabuk 71491, Saudi Arabia; aa-albalwi@ut.edu.sa

**Keywords:** cerebral palsy (CP), children, family-centered care, MPOC, health care services, Saudi Arabia

## Abstract

Family-centered care is widely considered as best practice in pediatric rehabilitation. We aimed to investigate parents’ perception of the family-centeredness of health care services for their children with cerebral palsy (CP) using the Arabic Measure of Processes of Care-20 (AR-MPOC-20). We also explored factors related to the child (sex, secondary impairments, and gross motor classification system level) and environment (family and residential region) that may influence the family-centeredness of services in Saudi Arabia. This was a cross-sectional study of 223 children with CP (age 6 months–18.2 years, M = 6.2 + 3.7 years) and their parents. Generally, parents perceived services as less family-centered. The lowest average score was for ‘Providing General Information’ (M = 2.9 ± 1.5), while ‘Respectful and Supportive Care’ had the highest average (M = 4.6 ± 1.8). Factors influencing the provision of family-centered care included being a female child and a mother’s educational level. In addition, all subscales of AR-MPOC-20 differed by region, *p* < 0.001, except for ‘Providing Specific Information’ which did not significantly differ by region *p* = 0.163. Clinicians should consider the families’ need for information regarding their children’s condition and available services, with special attention to the mothers of female children and mothers with low levels of education.

## 1. Introduction

Cerebral palsy (CP) is a neurological disorder characterized by impairments in movement and posture development that arise from non-progressive damage to the growing brain during the fetal or newborn stages of life [1,2]. CP is a prevalent neurodevelopmental condition in childhood, with a birth prevalence of approximately 1.6 per 1000 live births in high-income nations [3]. In Arab-speaking countries, the estimated prevalence is slightly higher, at 1.8 per 1000 live births and 2.34 per 1000 individuals specifically in Saudi Arabia [4,5]. Chronic childhood disability is a significant global issue that presents itself in several forms, including delayed motor development, musculoskeletal complications, and associated conditions such as epilepsy, visual impairment, hearing, and speech impairment [6,7,8].

The family plays an important role by fostering a supportive environment for children with CP and, thus, it is necessary to consider the family’s needs to improve a child’s life [9]. Currently, family-centered care (FCC) is widely recognized as the most effective approach in pediatric rehabilitation, particularly for children with neurodevelopmental disabilities [10,11,12]. FCC is an approach to health care that emphasizes partnership with the parents in the care of their child. This approach encourages the family’s active participation in recognizing the requirements and key concerns of their child’s care, and subsequently strategizing interventions tailored to those needs [10,12,13,14,15,16]. The basic principles of FCC include treating families with respect; parent–professional partnerships; effective communication; and providing treatment choices to enable families to make informed decisions [12,13,14,17,18]. Evidence has shown the beneficial outcomes of FCC on child developmental skills, service satisfaction, and the quality of life and psychosocial wellness for parents and children with disabilities [10,13,17,19,20,21,22,23,24].

The provision of FCC may be influenced by many factors related to the child, family, or environment. Previous research has shown that the extent of the family-centeredness of care is associated with the parents’ educational level and family income/socioeconomical status, but not related to a child’s secondary impairments [15,16].

In Saudi Arabia, the recent Saudi health care transformation, under the Kingdom’s vision 2030, has adopted the new Model of Care aiming to promote public health [25]. The emphasis of this model is on enhancing health care services through continuous improvement, which is centered around enriching the experience and satisfaction of the beneficiaries in alignment with the most effective strategies. However, the process of health care service delivery for the most common pediatric physical disability, CP, is yet to be explored. It is unknown whether the concept of FCC is implemented in the health system. Since FCC is considered to be the best practice in pediatric rehabilitation [10,12], it is important to establish the extent of the use of FCC in pediatric health care in Saudi Arabia for children with CP. Furthermore, identifying factors associated with the use of FCC may help in improving service delivery for children and their families.

The Measure of Processes of Care (MPOC) is an instrument commonly used to assess the parents’ perception of the family-centeredness of service delivery in pediatric rehabilitation [26,27]. The MPOC was translated into Arabic [28] and, therefore, it would be the most appropriate tool to use in Arab-speaking countries, such as in Saudi Arabia, for assessing parents’ perception of the family-centeredness of services in pediatric settings.

This study aimed, therefore, to examine parents’ perception of the family-centeredness of health care services for children with CP across Saudi Arabia as well as to explore the factors related to the child and environment (family and residential region) that may influence the family-centeredness of services. We hypothesized that potential child factors that may be related to the process of care are gross motor functional limitations, secondary impairments, and sex. In addition to the aforementioned, the potential environmental factors include parental education, family income, and residential region.

## 2. Materials and Methods

### 2.1. Study Design and Ethical Consideration

This research was part of a nationwide cross-sectional study of children with CP and the services provided to them in Saudi Arabia, which was conducted from February to August 2022. The study was conducted in the period after the COVID-19 outbreak. In this period, restrictions were eased, and all health care workers were vaccinated. Therefore, there were no major changes in health care service provision compared to the pre-COVID-19 period. The study procedure complied with ethical approval requirements in accordance with the regulations and guidelines established by the National Committee of Bioethics (NCBE). Approval was obtained from the local ethics committee of the University of Tabuk (UT-175-39-2022).

### 2.2. Participants

A convenience sample of children (age range 6 months–18.2 years) diagnosed with CP by their pediatric neurologist and their parents (N = 223) from 14 cities (in 8 out of 13 provinces) in Saudi Arabia were included in the study. Respondents were 81.6% mothers, 16.5% fathers, 0.9% both parents, and 0.9% others (aunts).

### 2.3. Measures

#### 2.3.1. Family and Child Information Form

A self-report questionnaire was utilized to gather demographic information regarding the child’s age, sex, and secondary impairments (e.g., visual impairments, hearing impairments, speech disorders, nutritional deficiencies, learning disabilities, attention deficits, epilepsy, respiratory conditions, musculoskeletal disorders, obesity, and pain problems). Furthermore, the educational level and family income of the parents were reported. Additional data collected included the geographical area of residence as well as services the child received in the past 12 months.

#### 2.3.2. AR-MPOC-20

The original version of the MPOC-56 is a validated and reliable self-report assessment tool that comprises 56 items in five subscales [27]. The shortened version, known as MPOC-20, maintains the same underlying framework as the MPOC-56, including five subscales. However, it achieves this with a reduced number of questions to be 20, while still demonstrating good levels of reliability and validity [29]. The MPOC-20 was translated into Arabic (AR-MPOC-20) and validated [28]; The MPOC-20 is a tool used to evaluate parents’ perspectives on the degree to which services provided to their children are centered around the family. The instrument consists of five subscales: (1) Enabling and Partnership, which comprises three items; (2) Providing General Information, consisting of five items; (3) Providing Specific Information about the child, including three items; (4) Coordinated and Comprehensive Care for the child and family, which includes four items; and (5) Respectful and Supportive Care, which consists of five items. The rating scale for all items ranges from 1, indicating no perception at all, to 7, indicating a high degree of perception. A rating of ‘not applicable’ is assigned a numerical value of 0. The score of each subscale is the mean score of the items comprising it. The MPOC-20 has been translated into Arabic (AR-MPOC-20) and its validity and reliability have been established (Cronbach’s α: 0.69–0.82) [28].

#### 2.3.3. The Gross Motor Function Classification System—Expanded and Revised (GMFCS) [30]

The GMFCS is a reliable and valid classification system consisting of five levels, specifically designed for children with cerebral palsy. Its purpose is to categorize their gross motor function, considering both their functional mobility capabilities and constraints. Level I represents individuals with minimal limits to their gross motor function, whereas Level V represents individuals with maximal functional restrictions and dependency in their mobility skills. Interrater reliability (kappa) was 0.55 for children aged less than 2 years, and 0.75 for those between 2 and 12 years old [28]. Trained pediatric physical therapists assessed and determined the GMFCS levels for the children involved in the study.

### 2.4. Procedure

Invitations to participate in the current study were presented to parents during their child’s regular physical therapy appointments. Physical therapists, working as research assistants (RAs), outlined the study for parents and explained its objectives and the process. Thereafter, they obtained written informed consent. The RAs conducted parents’ interviews and completed the child and family information form as well as the AR-MPOC-20. Although AR-MPOC-20 is a self-administered survey, the decision was made to conduct interviews with parents in order to provide them with the option to ask questions if needed. RAs were not the child’s treating physical therapist, which reduced social desirability or courtesy bias. Prior to the start of data collection, a training workshop with a duration of two hours was provided to the RAs to explain the measures and procedures. Finally, the RAs determined the child’s GMFCS level.

### 2.5. Data Analyses

The GMFCS was split into levels I to III (ambulatory with or without limitations) and levels IV to V (non-ambulatory). The total number of parent-reported impairments for each child was calculated. The eight provinces of residence were grouped into five geographic regions: north, south, central, east, and west. The highest level of maternal and paternal education was grouped into intermediate or less, high school and college, and bachelor or higher. The family monthly income (in Saudi Riyal) categories were >15,000; 10,001–15,000; 5000–10,000; and <5000.

For data analysis, SPSS Version 25 was used (SPSS Inc., Chicago, IL, USA). Descriptive statistics were utilized to describe sample characteristics. These statistics included frequencies for categorical data and means and standard deviations for continuous data. Then, the AR-MPOC-20 subscale means and standard deviations were calculated to determine differences in the AR-MPOC-20 subscale scores by child, parents, and regional variables; an independent t-test (for dichotomous predictor variables) and a one-way independent ANOVA (for predictor variables with more than two categories) were used. Bonferroni correction was applied to adjust for multiple comparisons. For this, a *p*-value of <0.05 was considered statistically significant a priori.

## 3. Results

### 3.1. Child and Family Characteristics

A sample of 223 children with a diagnosis of CP (age range 6 months–18.2 years, M = 6.2 ± 3.7 years) and their parents were included in the study. Table 1 shows the characteristics of the participating children and their parents. The most common service used in the past 12 months was physical therapy, followed by pediatric neurology.

Generally, the whole range of response options for each item of the AR-MPOC-20 was used by respondents (1, the lowest perception of FCC, to 7, a high degree of perception). The items ‘Provide enough time to talk so you don’t feel rushed’, ‘Treat you as an equal rather than just as the parent of a patient’, and ‘Fully explain treatment choices to you’ had the highest average scores (4.75, 4.59, 4.58, respectively), with 27.8, 26.5%, 25.1% and of parents rating these items as “to very great extent”. On the other hand, the items ‘Provide you with written information about your child’s progress’, ‘Provide you with written information about what your child is doing in therapy’, and ‘Give you information about the types of services offered at the organization or in your community’ had the lowest average scores (1.80, 2.38, 2.85, respectively), with 45.3%, 36.8%, and 31.8% of parents rating these items as “not at all”.

### 3.2. Parents’ Perception of Family-Centered Care

Total scores for each of the five subscales of AR-MPOC-20 are shown in Table 2. The subscale ‘Providing General Information’ has the lowest average score (2.9 ± 1.5), while the ‘Respectful and Supportive Care’ subscale has the highest average score (4.6 ± 1.8).

### 3.3. Factors Associated with Scores on the AR-MPOC-20

Table 2 also shows the scores of the AR-MPOC-20 scales in terms of the characteristics of the child, parents, and region. The scores of the AR-MPOC-20 scales did not differ by GMFCS levels, the number of secondary impairments a child has, the father’s educational level, or family income. However, parents of a female child had significantly lower scores on the Providing General Information subscale (t (154.3) = 2.7, *p* = 0.009) than parents of a male child (mean difference, 0.611, BCa 95% CI [0.14, 1.07]). In addition, the mother’s educational level had a significant effect on the Providing Specific Information subscale. Mothers with a bachelor degree or higher tended to score this subscale or item significantly higher than mothers with a high school or college educational level, F(2, 154) = 4.58, *p* = 0.012. Four of the five subscales of the AR-MPOC-20 differed by geographic region: Enabling and Partnership, F(4, 152) = 19.30, *p* < 0.001; Providing General Information, F(4, 152) = 12.02, *p* < 0.001; Coordinated and Comprehensive Care, F(4, 152) = 20.08, *p* < 0.001; and Respectful and Supportive Care, F(4, 152) = 20.93, *p* < 0.001. However, Providing Specific Information scores did not significantly differ by region, F(4, 152) = 1.66, *p* = 0.163.

## 4. Discussion

This study is the first study which has sought to investigate the adoption of FCC in health care service delivery for children with CP in Saudi Arabia. Parents of children with CP in Saudi Arabia perceived the family-centered behaviors of service providers to be low, ranging between 2: ‘to a very small extent’ and 5: ‘to a fairly great extent’. The factors affecting the parents’ perception of FCC were the child’s sex, mothers’ education, and residential region.

The parents’ ratings of FCC in this study were lower than the ratings in previous research [14] and thus, this low rating may indicate that FCC is still not effectively implemented in Saudi Arabia. Qohal and Kaddaf [31] identified obstacles that limit the implementation of FCC in pediatric nursing practices in Saudi Arabia. These constraints include inadequate staffing, insufficient time to establish trust and rapport, limited time for care negotiation, and language barriers which prevent effective communication with families. In addition, Alkhaibari et al. [32] conducted a systematic review to explore the existing research on the practice of patient-centered care in the Middle East, including Saudi Arabia, and the North Africa region. The review encompassed research conducted on both children and adults, revealing that there exists a degree of endorsement for the implementation of patient-centered care, particularly FCC, within the Middle East and North Africa region. However, it is important to note that this support is rather restricted in scope. Hence, it is imperative to emphasize the necessity of further endeavors in order to establish a family-centered approach to pediatric health care in Saudi Arabia.

It should be noted that this study was conducted in the post-COVID-19 period. Although, in Saudi Arabia, in this period all restrictions were eased, and all health care workers were vaccinated, we are not sure if the process of health care delivery was affected by the COVID-19 outbreak. More research is required to compare health care delivery in the pre- and post-COVID-19 periods. The pressure on the health system, the dismissal of many workers, and the economic crisis are all factors that may have affected the process of health care delivery.

The highest score of all the items of the AR-MPOC-20 in this study was for the Respectful and Supportive Care subscale. Previous studies [15,16,20] have also shown that the highest score was in this subscale as well. However, the lowest scores among the five subscales were for scales related to the provision of general and specific information. Previous research on FCC has shown a lack of this information [14,15,16,19,20,33,34,35]. This lack of information exchange is continually arising in almost all studies examining FCC. It is crucial to examine the factors limiting the provision of needed information to families. Health care service providers are urged to give enough time for parents to express their concerns and get their questions answered. An information board or a website where parents can find answers to their general questions about the resources/services offered by a center or community may help to improve service providers’ provision of general information [20]. Indeed, the provision of the needed information empowers families, enables them to make decisions, and decreases their feelings of anxiety and stress [12,36].

Factors affecting the parents’ perception of FCC included the child’s sex, mother’s education, and residential region. Other child and parental factors did not significantly affect the perception of FCC. Interestingly, parents of female children had significantly lower scores on the Providing General Information subscale, which serves as a unique finding in our study. As previous research did not report child sex as a determinant of FCC [15,16], cultural differences may be a potential explanation for our findings. Arab populations are usually overprotective toward female children and are more sensitive to their needs. However, more research is needed to further explain these findings. As was suggested earlier, the provision of easily accessible educational material such as information boards or a website may help in providing the general information needed by families.

The mother’s educational level significantly affected scores on the Providing Specific Information subscale. Mothers with a bachelor’s degree or higher tended to score this section significantly higher than mothers with a high school or college education. A Turkish study also reported the significant effects of a mother’s education on MPOC-20 scores [15]. The authors reported that mothers with lower educational levels had lower scores on Enabling and Partnership, Coordinated and Comprehensive care for the child and family, and Respectful and Supportive Care. Although the affected subscales in the Turkish study are different from our study, both findings indicate that health care providers need to be attentive to mothers with less education and provide support and information when needed. These mothers may be reluctant to express their concerns, and therefore health care providers are urged to help them communicate any anxieties. In addition, as Qohal and Kaddaf [31] reported, language barriers may be an important factor affecting information exchange. In Saudi Arabia, many non-Saudi health care professionals use English and are not proficient or fluent in Arabic, which is the official language in Saudi Arabia. Mothers with lower educational levels usually do not understand English well and therefore may not recieve specific information about their children’s progress and treatment options.

Four of the five subscales of the AR-MPOC-20 differed by geographic region, except for Providing Specific Information scores. One would expect the Providing Specific Information subscale to be consistently low in all regions and, therefore, to not differ by region. However, the differences in the other subscales by region may indicate differences in the health care delivery culture in different centers across Saudi Arabia. As FCC is still not well implemented in Saudi Arabia, the reasons for the discrepancies in the family-centeredness of services across regions need to be determined and addressed. In Saudi Arabia, the transformation of health care and the new model of care are anticipated to implement systematic assessments of population’s needs and the effectiveness of the health system to optimize the distribution of resources and provide the results that people require [25].

Interestingly, the parents’ perception of FCC was not associated with the child’s impairments or their GMFCS level. These findings were also reported by previous studies [15,16,20,21] and indicate that the caregivers’ approach to service delivery does not change according to the child’s health condition. Indeed, the service type and intensity may vary depending on the severity of the disease (impairment and motor functional limitations), however, our study examined only the process of service delivery and the extent of it being family-centered.

### 4.1. Implications

1. Families of children with neurodevelopmental disabilities still express a need for more general and specific information regarding their children’s condition, treatment options, and available services.
2. Service providers should be attentive to mothers of female children with disabilities and mothers with lower levels of education.

### 4.2. Limitations of the Study

The main limitation of this study is that the findings may not be generalized to the northern and eastern regions due to the fact that the regions were less represented, as we could not recruit an appropriate number of RAs in the area. Nevertheless, this study still included many provinces in Saudi Arabia that represent the five geographic regions. In addition, it should be noted that this study explored only the family’s perception of FCC. Therefore, our results do not provide information on the professionals’ training or motivation to apply FCC. Indeed, professional training and motivation is important to facilitate the implementation of FCC in health care centers providing services for children with disabilities. Therefore, we recommend further research to explore this area. It is also worth noting that this study used parent-reported information, including about the child’s impairments. These impairments may not be the same as the impairments in the medical file of the child. Another source of potential bias is the use of interview rather than self-reporting. However, we decreased the risk of this bias by having RAs who were not the child’s treating therapist. We also emphasized to parents that their participation in the study would not affect the provision of services to their child. Only two participants were ‘both parents’, therefore, we do not think that parents’ candid responses were affected by having both parents in the interview.

## 5. Conclusions

Our multisite study suggests that the implementation of FCC is still not up to the required level of the health care service provision for Saudi children with CP and their families. Parents of children with CP in Saudi Arabia perceived the health care services offered to their children as less family-centered. Thus, health care providers across Saudi Arabia serving children with disabilities are urged to enforce the implementation of the principles of FCC in their practice.

## Figures and Tables

**Table 1:** Child and family characteristics (N = 223).

Characteristic	n (%)	Characteristic	n (%)
Region		Sex	
South	51 (22.9)	Male	131 (58.7)
Center	49 (22.0)	Female	92 (41.3)
East	28 (12.6)	^1^ GMFCS level	
West	88 (39.5)	I-III	123 (55.2)
North	7 (3.1)	IV-V	100 (44.8)
Mother’s educational level		Number of comorbidities	
Intermediate or less	42 (18.8)	0	34 (15.2)
High school and college	97 (43.5)	1–2	80 (35.9)
Bachelor or higher	84 (37.7)	3–4	61 (27.4)
Father’s educational level		≥5	48 (21.5)
Intermediate or less	36 (16.1)	Use of services in the past 12 months	
High school and college	111 (49.8)	Orthopedic surgeon	88 (39.5)
Bachelor or higher	76 (34.1)	Pediatric neurologist	135 (60.5)
Household monthly income		Physical therapy	215 (96.4)
>15,000	36 (16.1)	Occupational therapy	76 (34.1)
10,001–15,000	48 (21.5)	Speech therapy	32 (14.3)
5000–10,000	97 (43.5)		
<5000	42 (18.8)		

^1^ GMFCS, Gross Motor Function Classification System.

**Table 2:** The Measure of Process of Care AR-MPOC-20 subscale scores * according to child, parent, and regional characteristics.

	Enabling and Partnership	Providing General Information	Providing Specific Information about the Child	Coordinated and Comprehensive Care for the Child and Family	Respectful and Supportive Care
	Mean (SD)				
Total scales	4.5 (1.9)	2.9 (1.5)	3.2 (1.6)	4.4 (1.7)	4.6 (1.8)
Sex					
Male	4.7 (1.9)	3.1 (1.6) ^a^	3.4 (1.6)	4.6 (1.7)	4.6 (2.0)
Female	4.1 (1.8)	2.5 (1.2) ^a^	2.9 (1.6)	4.4 (1.6)	4.7 (1.8)
GMFCS level					
(1) I-III	4.6 (1.7)	2.9 (1.5)	3.3 (1.7)	4.6 (1.6)	4.9 (1.8)
(2) IV-V	4.3 (2.0)	2.8 (1.5)	3.1 (1.6)	4.3 (1.8)	4.4 (2.0)
Number of impairments					
0	4.9 (1.7)	3.4 (1.3)	2.7 (1.1)	4.8 (1.6)	5.1 (1.5)
1–2	4.2 (1.9)	2.7 (1.5)	3.0 (1.6)	4.3 (1.7)	4.5 (1.9)
3–4	4.7 (1.7)	2.9 (1.6)	3.5 (1.7)	4.5 (1.6)	4.5 (1.8)
≥5	4.4 (1.9)	2.9 (1.6)	3.5 (1.8)	4.3 (1.9)	4.5 (1.8)
Mother’s educational level					
Intermediate or less	4.3 (2.0)	2.5 (1.0)	3.1 (1.5) ^a^	4.4 (1.6)	4.3 (1.8)
High school and college	4.4 (1.9)	2.9 (1.6)	2.8 (1.5) ^a^	4.2 (1.8)	4.6 (1.9)
Bachelor or higher	4.7 (1.8)	3.1 (1.6)	3.6 (1.8) ^a^	4.6 (1.6)	4.7 (1.7)
Father’s educational level					
Intermediate or less	4.8 (2.1)	2.8 (1.4)	3.1 (1.6)	4.8 (1.9)	4.7 (2.0)
High school and college	4.5 (1.8)	2.9 (1.4)	3.2 (1.5)	4.5 (1.6)	4.6 (1.7)
Bachelor or higher	4.3 (1.9)	3.0 (1.6)	3.2 (1.7)	4.2 (1.8)	4.5 (1.9)
Household monthly income					
>15,000	4.3 (1.9)	2.9 (1.6)	3.4 (1.7)	4.3 (1.8)	4.6 (2.0)
10,001–15,000	4.3 (1.8)	2.6 (1.3)	3.2 (1.7)	4.3 (1.7)	4.4 (1.8)
5000–10,000	4.4 (1.9)	3.1 (1.6)	3.1 (1.6)	4.4 (1.7)	4.6 (1.8)
<5000	5.1 (1.8)	3.0 (1.5)	3.2 (1.5)	4.6 (1.6)	4.9 (1.7)
Region					
South	3.8 (1.7) ^a^	2.4 (1.1) ^a^	3.3 (1.4)	3.9 (1.4) ^a^	4.1 (1.9) ^a^
Center	4.8 (1.6) ^a^	2.3 (0.8) ^a^	2.8 (1.7)	4.5 (1.6) ^a^	4.8 (1.5) ^a^
East	2.5 (1.7) ^a^	2.2 (1.3) ^a^	2.7 (1.6)	2.8 (1.8) ^a^	2.6 (1.7) ^a^
West	5.2 (1.5) ^a^	3.7 (1.6) ^a^	3.5 (1.7)	5.3 (1.4) ^a^	5.4 (1.4) ^a^
North	5.5 (1.8)	4.9 (1.3) ^a^	4.3 (1.4)	4.6 (1.7)	5.3 (1.3) ^a^

* The rating scale ranges from 1, indicating no perception at all, to 7, indicating a high degree of perception. ^a^ Significant after Bonferonni correction (*p* < 0.05).

## Data Availability

Restrictions apply to the datasets; the datasets presented in this article are not readily available due to ethical and patient privacy considerations. Requests to access the datasets should be directed to the corresponding author, Ahmad Alharbi.
